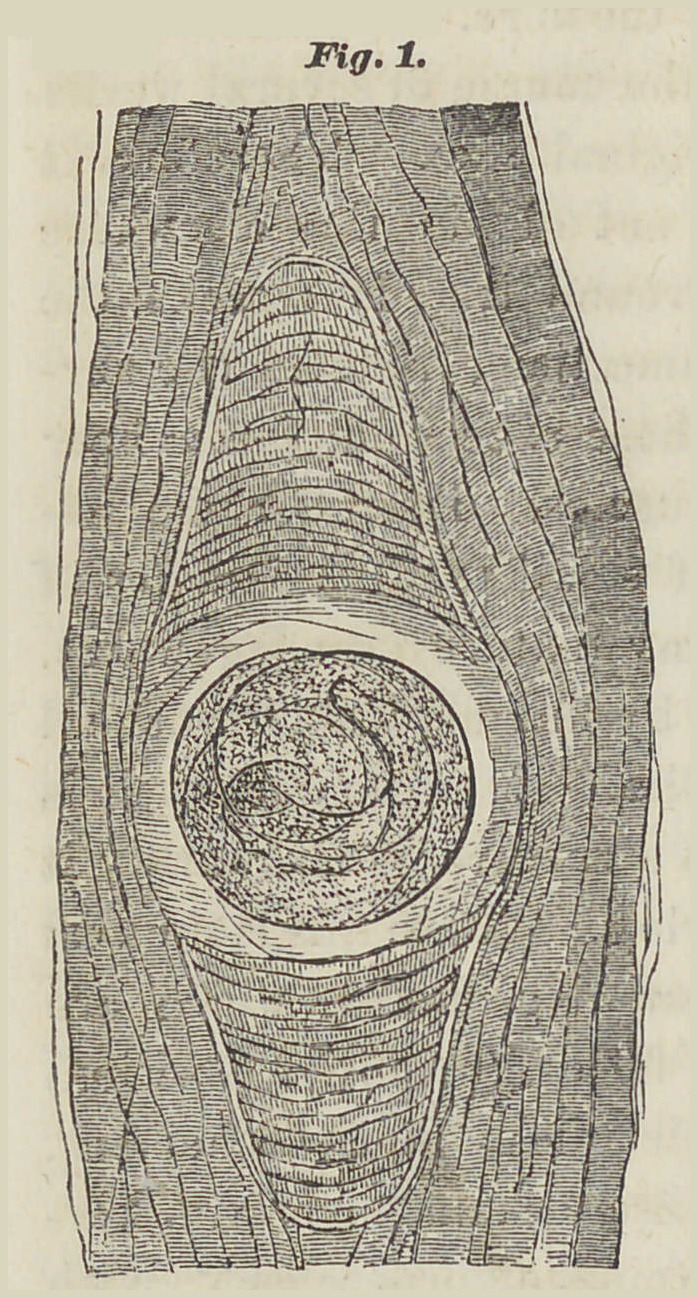# The Life of the Trichina

**Published:** 1868-10

**Authors:** Rudolph Virchow

**Affiliations:** Prof. University of Berlin


### Original Communications.

### THE LIFE OF THE TRICHINA.

BY RUDOLPH VIRCHOW, M.D.PH.D., PROF. UNIVERSITY OF BERLIN.

Translated by Rufus King Browne, M. D.

### INTRODUCTION.

The observations, increasingly frequent of cases of disease, and even death, which have been caused by a microscopic worm called Trichina, have periodically attracted an extended public attention and also caused in many localities well founded alarm of the danger involved in food. Numerous enquiries by physicians and laity, whom it would take too much time to answer individually, decided me, in order to enlighten the public mind, to whom scientific means for the investigation are wanting, to bring together the most essential facts, and by illustrations from nature to give the public the means of an understanding of them. I wish it understood that I only report facts, and where these were before wanting I have supplied them.

---

The Trichina as it appears in meat is a microscopic animal, or, in other words, it is to the naked eye, under ordinary circumstances invisible. Still, it is not to be thought, as I have heard here and there, that it is the same as infusoria, of which some people have the idea, that they are everywhere to be found in a drop of water, and in every portion of the air. Pure water, pure air, and pure meat do not contain infusoria nor any other species of animal; only putrid fluids and organic parts may contain infusoria, but this is not always the case. With these animals more or less widely spread, the Trichina has nothing in common; it belongs to another class of animals, that of the worms, and is only found under peculiar circumstances. Moreover, it is not only the small size which makes it invisible to the naked eye, for we can see other bodies of the same size very well. Quite frequently it attains a length of from ⅓ to ½ a line. But its body is in a high degree transparent, which is explained by the fact that its single parts and organs are very little developed. If the body was opaque and reflected the light, one with good eyesight and careful observation could see the animal very easily. As it is, one can only by a combination of favorable circumstances see any signs of it. If we bring the Trichina, the body of which is curled up, and therefore occupying its least space, in a drop of water, on a plate of glass, and place this upon a black surface, we see a whitish point. More can not be seen, and therefore it is impossible to recognize that this point is an animal.

Frequently the animal in the muscle is enclosed in a peculiar capsule, a kind of sack without opening, a so-called cyst. This capsule has frequently a considerable size and thickness. So long as it is tender and not fully developed, the naked eye can scarcely see it. If it become more developed and increases in density and thickness, and lime salts are deposited in it, it presents more obstacles to the transmission of light, becomes opaque and appears as a small whitish body.

Thirty years ago these bodies attracted the observation of physicians. An English anatomist, Hilton, seems to have been the first to investigate them. He supposed them to be animal structures, but did not at once recognize the worm they contained. It was in 1835 that the celebrated zoölogist Owen, described this worm and gave it the name Trichina

Spiralis, because its body is small as a hair, (from Trichos) and coiled up spirally.

A number of other observers in England, France, Germany, Denmark and North America announced the fact that Trichina in these capsules had been found in persons of these several countries. In animals the observed cases are very few. They were found in the cat,[1] crows, blackbirds, hawks and other birds as well as in moles[2] and pigs.[3] But it is not certain that all so found belonged to the same species, or whether Trichina Spiralis has not been confounded with another species, Trichina Affinis.[2]

Although these observers disagreed among themselves, whether the capsule which envelopes the animal is either mostly or in part, a part of the animal, it was taken for granted to consider the whole as one, and to pronounce only such meat as containing Trichina in which the whitish bodies could be observed with the naked eye. This conception could only be correct provided that the capsule was an egg shell, for if the animals had developed from eggs in the spot where they were found, of course the capsule must have existed from the beginning. This was, however, in a high degree improbable. Later it has been ascertained by a more special investigation, that there is no question of an egg in the case.

With the certainty of this fact,[1] the capsule, of course, has another significance. Either it was a secretion, a product of the animal, or a formation from the part of the human body in which the animal lodges. There was in its history a time when there was no capsule, and the animal consequently was free. But nobody had previously seen them free in the human

---

1. C. F. Gurlt, appendix to the first part of his book of pathological anatomy of the domestic animals. Berlin. 1849. pp 144.

2 Julius Fogel. Path. Anat. of the Human Body. Leipsig. 1845 pp 422. Herbst on the Nature and Spreading of the Trichina Spiralis Data (Nachricthen) from the G. A. University and the Royal Soc. Sci Gœttingen. 1852. pp 183.

3. Zencker on Trichina Disease of Man. Virchow Arch. for Path. and Phy. and for Clinical Medicine. Vol. XVIII. pp 561.

body. The first observation of this kind was made by Zencker,[1] Dresden, in a case of Trichina disease which resulted in death, and which has since become of great importance, and to which I will again allude.

We now know that a considerable time, at least two months, is necessary to produce a complete capsule, and that any man or animal who lives so long that the Trichina in them becomes enclosed, are nearly past all danger. We may therefore safely say that all observations of Trichina which up to the year 1860 have been made, were of cured cases.

It is, therefore, easy to understand that more and more the conviction spread that the Trichina was a harmless animal. A mere curiosity. Practical physicians ceased to have any interest in it, and left the anatomist and zoologist to pursue the subject as a purely scientific one.

Certainly, however, it has a great scientific interest, and to this circumstance it is due that in this case the old saying of the stone that the builders rejected, and which became the corner stone was exemplified. What was extraordinary was where the Trichina came from, and how it came into the muscles of living persons. As one could not discover any facts which pointed to propagation; for there was neither found any young, or eggs or developed genital organs.

Such cases, had been easily disposed of not long ago by the theory, that there existed a *spontaneous* generation (epigenesis, equivocal, or spontaneous generation.) Among the people generally as well as a certain number of investigators, the opinion was still held that from certain substances, especially from excrementitious and putrid things, living animals especially vermin would arise. In this class was included the intestinal vermiculæ, because it was not understood how they arose in the bodies of living animals, if they had not as supposed been created within them. Respecting the Trichina this idea did not seem so far wrong, since they were to all ap-

1 Zenker on Trichina Disease of Man. Virchow Arch. for Path. and Phy., and for Clinical Medicine. Vol. XVIII.

pearance entirely sexless, and devoid of all qualities which the course of generation pre-supposes. In addition is the circumstance that they are found in immense numbers, for in some cases millions of Trichina are found in a single person. Such a number of any other of the intestinal entozoa have never been found in any one person. Was it not supposable that the Trichina arose from some impurity in the system?

The Trichina in these relations resembles certain worms, especially the cysticercus which are frequent in pigs, and are also found in men. The cysticercus was different from the Trichina in this, namely, that they are much larger, for the Trichina even if the capsules be included, form only a small white point or thin line. The cysticercus attains the size of a pea, and sometimes of a small cherry or bean. To mistake one for the other is not possible even for the inexperienced. The cysticercii are also sexless, they have no eggs and are often found in great numbers. They are enclosed in the flesh, and are in many respects like the Trichina, and even their origin by spontaneous generation seemed the most probable.

The best investigators of the last century, especially the distinguished Pastor Goetze, of Quedlinburg, noticed that the cysticercus had a great likeness of structure, with the head of the tape-worm, and these investigators had the two in one genus, that of the Teniæ. Still they considered them to be species of the same genus, which compared with each other as donkey and horse, dog and wolf.

Only further investigations of recent times led to the idea that the relation of these was nearer, and that the cysticercus was a real tape-worm, developed under different conditions from the latter. But the immediate experience of Kuchenmeister, from actual experiment, showed that this conjecture was not wholly true. He found that the cysticercus of the muscle when it is eaten developes in the intestine to a tape-worm. It has, therefore, lived for a time in the state of the cysticercus, and subsequently assumes that of the tape-worm.

The question is, how the worm came into the first state and into the muscle. In its tape-worm state it produces on its posterior extremity by growth and casting off, new beings, of which each of itself not only contains eggs but even produces living young. These, however, leave the egg state only after they have been discharged from the intestine, and thus, either by eating or drinking are taken into the body.

As soon as they enter the stomach the shell opens and the young, then microscopic animals are freed, pierce the intestinal wall and by active and passive movements, reach different parts of the body, and there develope into cysticercii. This is a long, and in a great degree chance development. The cysticercus is first eaten with the meat containing it, before it changes into a tape-worm, and from this in its single parts is generated eggs, and these must again be taken into the system to become embodied in the flesh and developed into a new being. It, therefore, not only changes location several times but a change of generation also takes place, for every member of the tape-worm is a representative of a different generation.

With these experiences the old doctrine of spontaneous generation of the intestinal worms was shaken to its foundation. If even so large animals as the cysticercus are produced regularly from generation to generation from eggs, to reach by peculiar wanderings the muscle from the intestines, it was easy to suppose that something similar to this, might take place in case of the Trichina. Certainty was only to be had by experiment.

Herbst, in Gœttingen was the first to institute such experiments. He found that afterwards animals which had been fed with meat containing Trichina had Trichina in their muscles. His experiments, however, were defective. In the first place it was not thereby determined, that the Trichina which had been fed were identical with those found in human beings. In the next place he had not been able to pursue the history of the case between the time when the Trichina entered the stomach, and that when they were found in the muscle.

Was there a change of generation? Were the Trichina in the intestine changed into another intestinal worm? Did they produce eggs? Or were the same Trichina which had been eaten found in the muscle?

More experiments with feeding especially by Kuchenmeister had no result, but he supposed that the Trichina changed in the intestine into another known intestinal worm, the Tricocephalus, and that the former was the immature state of the latter.

This conjecture, at first, seemed to be confirmed by Leuckhart in Giessen who had formerly found, after feeding Trichina meat to mice, free Trichina in the intestinal mucus.

On the 28th of September, 1859, he communicated to the Parisian Academy, an account that he had succeeded in producing Tricocephalæ, in great numbers from Trichina.

I had, at the same time, arrived at another result. In a dog fed with Trichina from a human body, which were encapsuled, I found, three and a half days after feeding, numerous free and full grown Trichina, which had attained to a perfect sexual development. I could distinguish male and female animals, and in the bodies of the latter I found numerous egg and germ cells.

On the first of August, 1859,[1] I made my first communication on the subject to the Medical Society in Berlin, and a more special communication in my archives.[2] At that time I showed that the capsule in which the animal was found in the flesh, was nothing else than a changed muscular fibre—a deteriorated primitive bundle, and therefore the animal had to enter the structure of the flesh.

These results have been verified by subsequent experiments of feeding, first by Leuckhart and myself, and also by Claus and others. Especially the case, already mentioned, observed by Zencker in January, 1860, gave to Leuckhart, as

1. Deutcher Clinik. 1859. pp 430. Compt. Rend., de l'Acad. des Sciences. Tome XII. pp 660.
2. Archive for Path. Anat. and Phy. Vol. XVIII. pp 342.

well as myself, new material for experiment. The former reported it in a large volume.[1] I communicated my later experiences first, in a short account in my archives, and again in a longer communication to the Paris Academy.[2] The main result of our experiments was that the muscle Trichina, (muskel-trichina) given in meat food changed in the intestine only in respect to growth, (Darm-trichina) and produces eggs and living young in itself, and that these living young, without leaving the animal, immediately pierce the intestinal wall, penetrate the body and lodge especially in the muscular fibres, and then become encapsuled to remain there until they are eaten.

The case of the Trichina is, in one particular, different from that of the tape-worm and cysticercus. The former when *first* eaten produces a new brood which immediately penetrates the body.

The former need not be eaten twice, like the latter, to produce a new brood that penetrates the body; the danger is, therefore much greater in the case of the former, for the latter never endanger life, while we know a number of cases in which death has been caused by Trichina. In other respects the muscle Trichina and the cysticercus resembled each other in the fact that not the same animal which is eaten penetrates the body, but the *new* brood generated in the intestine enters the muscles.

After this general review of the development of our knowledge of the Trichina, I will proceed to state the main particulars more precisely.

1. How do we recognize the Trichina in the meat? In the first part of this treatise I have shown that though we can see an isolated worm under circumstances already mentioned, the Trichina itself cannot be seen in the meat with

---

1. Leuckhart, Investigations of Trichina Spiralis. Leipzig and Heidelberg. 1860.
2. Virchow's Arch. 1850. Vol. XXXIII. pp 535.
3. Compt. Rend. Tome. LI. pp 13. Compare Gaz. Med. de Paris. 1860, No. 28, pp 404.

the naked eye, and that what we can so see are only the capsules. Let us, therefore, consider the latter.

After a young Trichina has wormed into a muscular fibre, it moves on apparently for a certain distance. In this process it breaks the finer constituent parts of the fiber, and by this, probably, partly destroys the fibre. But there is no doubt that it even devours part of the fibre. It has a mouth, esophagus, and intestines, and in the course of several weeks grows to 30 or more times its original size. Therefore, it must take nourishment, and can not obtain this elsewhere than from the elements which surround it. It attacks the muscular substance, and, at the same time, irritates the surrounding parts. To understand these effects one has to bear in mind the construction of the muscles. Even for the naked eye, muscular flesh consists of small parallel bundles of fibres, held together by a very filmy texture (Bindegewebe). Each fasciculus can be seperated, by fine needles, into small bundles, and these again into fibrillæ. Microscopically it is seen that even the single fibre is a structure. Outwardly it has a structureless cylindrical covering. Within this is the real flesh elements, which consist of small granules, which are lengthwise in the form of minute fibres, (primitive fibrillen) and breadthwise in form of minute disks (fleischscheiben). Between them, at small distances, are certain parts with kernels, the so-called muscular bodies (muskelkörperchen). With a strong magnifying power, the single fibre shows itself as a very complex structure, a bunch of small fibres (prim. fibillen) which are held together by a common covering, and this is the reason why German anatomists have given to the fibre the name of primitive bunch.

The destructive effect of the Trichina is mostly on the flesh stuff (fleischscheiben) namely, the primitive fibres, and disks. These disappear within the fibre and the latter shrinks in proportion. The irritating effect appears most on the covering, and on the muscular bodies, particularly the kernels, and most strongly on that part the animal occupies. The covering thickens, the kernels of the muscular bodies increase; these bodies also grow, and between them a heavier substance is deposited, and bye and bye a denser mass forms around the animal in which can be seen the outer covering, and the interior enlargement. The more the animal grows the more it rolls up, curling in its extremities, and lies like a spiral coil.[1]

*Fig. 1.*

Generally this spiral, in a certain part of its circumference, immediately touches the covering, while over and under this place, lies the mass which proceeds from the enlargement of the contents. Where they touch, the capsule it is from the first thicker and less transparent.

These processes take place mainly in the third or fifth week after their emigration. From that time the density of the capsule increases, but the inner part more so than the covering. The middle part of the capsule, where the coiled animal reposes, under a moderate magnifying appears as a clear egg-shaped mass, (observe the figure 1) in which the animal is distinctly visible. Above, and beneath this spot, there are, usually, found two appendages which appear darker by transmitted light, by reflected light appear whitish. These tapering appendages have rounded off ends. Frequently they resemble in form the inner canthus of the eye. These appendages differ in length, and sometimes a similar difference in length exists likewise in the end of the same capsule.

In some cases these appendages are entirely absent, and

1See Fig. 1.

the capsules are either of a simple oval form, or rounded off at the corner, or are indented.

Those parts of the muscular fibre above them dwindle, but in the surrounding bindegewebe one sees sometimes inflamed excrescence, with the development of new vesicles.

The above changes require a lapse of months for their development. If such meat is examined with the naked eye, nothing peculiar is discoverable. If a small particle of it is covered with acetic acid or potash, by which addition they become transparent, small whitish opaque points on the ends of the capsules are visible. But if these are few in number, they are not so characteristic that we can, without the microscope, recognize the dangerous condition of the meat. We must be careful to guard against mistakes. Particles of fat frequent in meat, sections of vessels,* nerves, tendons, and parasitical deposits may present the same appearance, and the presence of the capsule only be distinctly seen, by a certain magnifying power.

The magnifying power need not be high. With a power of 10 or 12 we can readily see the relation, and distinguish both the capsule and animal. A somewhat higher power, as 50 or 100, is, however, more desirable, inasmuch as it precludes the liability to deception. If a still longer time elapses after the animal has migrated, other changes occur in the capsules, the most usual of which is, that lime salts are deposited, when the capsules are called "chalky."

Formerly, it was believed that the animal itself changed to chalk, but this is scarcely ever the case. Generally, this change to chalk begins in the thickened interior, while the exterior remains unchanged. The lime salts appear as very minute granules which, by reflected light, appear white, and with transmitted light appear dark, shady, or altogether black, and if the lime increases, it eventually covers the

---

*Translator's Note.—The word *gefasse*, synon. vessel, is used here by the author.

whole animal, and we cannot distinguish the latter under the microscope, even if it be entire. It is then encased as by an egg-shell.

If the human body, into which the Trichina penetrates, is well nourished, another change occurs. Around the capsule, and particularly around their appendages appear fat cells.[1] When this deposit of fat attains a certain size it forms around the capsule a lump, which marks the location of the capsule more plainly than it is by the chalky deposit, for from the moment the former attains a certain magnitude, the capsule is visible to the naked eye as a white spot, and this is the condition to which all the earlier observations relate.

In figure 1, this condition of a human muscle is shown. Upon the red surface, striped lengthwise by its bundle of fibres, as it appears to the naked eye, are a certain number of globular or egg-shaped points, on which, by very careful observation, can be distinguished the less opaque centre, indicating the position of the worm (see fig's. 1 and 2). These figures are from a cured case, in which the deposit of lime occupied merely the appendages. When this wholly covers the capsule, the latter becomes more palpable.

If acetic or hydrochloric acid be added to these white points they nearly disappear. But these acids produce precipitates from the meat juice, and cause the entire surface of the meat experimented upon to assume an indistinct and murky appearance. The best plan is to cut out small particles with fine scissors, teaze them out with fine needles, and thus separate the capsule from the meat itself. When this is done on a plate of glass, on a dark surface, one can see the capsule as white grains, and mark the dissolving effect of the acids.

This mode of investigation is best done, not by the naked eye, but by the microscope, nevertheless, to those who are experienced, the capsule in this chalky state is so characteristic that the confounding it with other appearances, is im-

1. In fig. 3 we see the fat cells around the appendages as vesicles.

possible. For investigation of meat in such a case, it is sufficient to carefully examine the meat and if white points be noticed, to add the acids, as before mentioned. If they are nearly dissolved by the acids no doubt remains. If they remain white, it is probable that they are particles of fat, or sections of nerve fibres, or similar structures. Nevertheless, we must remember that lumps of fat may exist near the chalk capsules, and that, therefore, the negative success of the experiment is less convincing than the positive. This is especially true in case there are few Trichina, because these are generally cured, the capsules being chalked and closely encased in deposits of fat. Moreover, the whole appearance is then less characteristic. It is self-evident that the investigation with the microscope alone furnishes a sufficient guarantee.

I now refer to a peculiar case. Some time ago, Meisner[1] found in most of the muscles of the mouse white stripes, visible to the naked eye, and which, on microscopic examination, were seen to be cylendrical tubes, each of which contained a number of long kidney-shaped bodies, of which it was doubtful whether they were of a parasitical nature, or formed a disease of the muscles.

Later, Von Heszling[2] found the same structures in the heart of the deer, and also, but more frequently, in that of the sheep. Von Siebold and Bischoff[3] observed them in rats. Lately, I received from Messrs. Dr. Grundler and Archidiaconus Ad. Schmidt, of Ascherslabeu pieces of pig's flesh, together with drawings representing the same structures.

By investigation, I have become satisfied that these are essentially the same as those found in the heart of the sheep, and I have no doubt that these are not animal products, but a parasitical structure; but, I am not determined whether, as

1. Meisner in the Report on the Proceedings of the Society for the adv. of Nat. Science, in Basle, 1843. pp 143. 2. Von Heszling. Pro. for Scientific Zoology. Vol. V. pp 196. 3. See the fig's in Von Siebold in part for Scientific Zoology. Vol. V. Plate X. Fig. 10–11.

Von Siebold believes, they belong to the vegetable world, or whether they are animal bodies. They bear a near resemblance to certain forms of zöosperm. Certainly the tubes which contain them present to the naked eye an appearance very similar to the capsules of the Trichina, and I mention them here, that mistaking them for the latter may be avoided.

Whether they are dangerous to life I cannot decide now, for we have made no observations about it. Further investigations may decide. Suffice it to say that these tubes differ from the Trichina, in this, that they have never been found encased in the lime deposit, and that the capsule does not pertain to the muscle, and that they do not contain worms, but only the minute egg or kidney-shaped bodies. This discovery shows clearly that the microscope only can determine the facts in any investigation of meat.[1]

---

1. The same. pp 201.

TO BE CONTINUED.

## Figures and Tables

**Fig.1. f1:**